# INBIA: a boosting methodology for proteomic network inference

**DOI:** 10.1186/s12859-018-2183-5

**Published:** 2018-07-09

**Authors:** Davide S. Sardina, Giovanni Micale, Alfredo Ferro, Alfredo Pulvirenti, Rosalba Giugno

**Affiliations:** 1 Department of Computer Science, University of Verona, Strada le Grazie 15, Verona, 37134 Italy; 2 Department of Mathematics and Computer Science, University of Catania, Viale A. Doria 6, Catania, 95125 Italy; 3 Department of Clinical and Experimental Medicine, University of Catania, c/o Dept. of Math. and Comp. Science, Viale A. Doria 6, Catania, 95125 Italy

**Keywords:** Protein interaction network, Network inference, Protein expression, Network algorithm

## Abstract

**Background:**

The analysis of tissue-specific protein interaction networks and their functional enrichment in pathological and normal tissues provides insights on the etiology of diseases. The Pan-cancer proteomic project, in The Cancer Genome Atlas, collects protein expressions in human cancers and it is a reference resource for the functional study of cancers. However, established protocols to infer interaction networks from protein expressions are still missing.

**Results:**

We have developed a methodology called Inference Network Based on iRefIndex Analysis (INBIA) to accurately correlate proteomic inferred relations to protein-protein interaction (PPI) networks. INBIA makes use of 14 network inference methods on protein expressions related to 16 cancer types. It uses as reference model the iRefIndex human PPI network. Predictions are validated through non-interacting and tissue specific PPI networks resources. The first, Negatome, takes into account likely non-interacting proteins by combining both structure properties and literature mining. The latter, TissueNet and GIANT, report experimentally verified PPIs in more than 50 human tissues. The reliability of the proposed methodology is assessed by comparing INBIA with PERA, a tool which infers protein interaction networks from Pathway Commons, by both functional and topological analysis.

**Conclusion:**

Results show that INBIA is a valuable approach to predict proteomic interactions in pathological conditions starting from the current knowledge of human protein interactions.

**Electronic supplementary material:**

The online version of this article (10.1186/s12859-018-2183-5) contains supplementary material, which is available to authorized users.

## Background

The understanding of the cell behavior and the characterization of the human tissues relies on both experimental and advanced information technologies. The release of The Cancer Proteome Atlas (TCPA) has provided proteomic expression data for 190 proteins in 16 cancer types using reverse-phase protein arrays (RPPA) technology [1]. This technique is able to measure hundreds of protein expression levels in many cancer tissues and makes possible the study of their differences and commonalities.

TCPA dataset contains phosphoproteins which provides information about the role of post-translational modifications (PTMs) such as phosphorylation in cancer. Other common PTMs include methylation and ubiquitination [2]. In general, these modifications affect the cellular processes by regulating protein-protein interactions (PPIs) being a remarkable key component in cell signaling, especially when dealing with cancer cells [3].

Extracting valuable information from proteomic data relies on the prediction and the analysis of their interactions. In this direction, network inference methods are widely used mainly on gene expression data. In [4], authors have gone beyond by proposing protein interactions inference, on 11 human cancers. For this purpose they used as reference model for existing interactions curated biochemical pathways stored in Pathway Commons [5]. However, pathways mostly do not contain direct protein interactions. Therefore authors infer such interactions by using Prior Extraction and Reduction Algorithm by specifying the distance of the shortest path that has to exist between two proteins within a pathway in order to consider those two proteins as interacting. Reference models are usually referred as gold standards. In this paper we propose a method called Inference Network Based on iRefIndex Analysis (INBIA), to infer protein-protein interaction networks from proteomic data, that attempts to overcome some of the limitations reported in [4]. More precisely, we overtake the bias in the knowledge base and the lack of context information for PPIs by considering iRefIndex [6] as gold standard for PPI networks inference [7]. iRefIndex is a consolidated database, which accurately integrates non-redundant PPIs from several sources by taking into account protein sequences and taxonomy [6]. We used 16 tissues data from TCPA and a set of 14 inference methods based on correlation, partial correlation, mutual information, and regression. The statistical correlation between protein expressions has important biological pros and cons because it may entail direct or indirect interactions [7], where the latter represents paths of pairwise directly connected proteins. Direct and indirect interactions may represent models of signal transduction, innate and adaptive immune signaling, cell cycle, metabolism, and DNA repair process.

We evaluated INBIA by comparing it with the method presented in [4], called PERA, in terms of true positive and true negative rates of inferred interactions considering both direct and indirect interactions. For each cancer type, we established the best performing methods. Moreover, we constructed predicted networks by assembling the most accurate results and associating consensus weights to interactions.

We evaluated the accuracy of predicted networks by annotating them in Negatome [8], and by comparing them with the tissue-specific PPI networks retrieved from TissueNet [9] and GIANT [10]. We also provide the functional and topological analyses of these networks, by reporting the different patterns of mutated genes and characterizing their diversities. Analyses showed that INBIA is a valuable resource to predict proteomic interactions in new pathological conditions starting from the current knowledge of human protein interactions.

## Methods

### Datasets

We downloaded data from TCPA [11] containing RPPA expressions of 190 proteins and phosphoproteins over 16 different cancer types (See Additional file 1: Table S1). The RPPA technology is similar to microarray for gene expression meaning that it can measure many proteins at the same time, but conversely it is based on specific and high-quality antibodies. Each cancer-related dataset has a different number of samples. Datasets are normalized by applying loading control for most of the tissues, whereas replicates based normalization is applied for *BRCA*, *SKCM* and *THCA*[1]. The normalization in RPPA dataset is needed because, like the western blot technique, each sample has different features such as cell number and membrane transfer efficiency. The loading control is a biological normalization procedure that relies on the quantification through specific antibody of another protein in order to compare their relative amount[12, 13].

The prediction of true interactions largely depends on the chosen gold standard. We use iRefIndex [6] as comprehensive and non redundant resource.

It includes 673,100 interactions covering proteins belonging to human and other species. Among these PPIs, we selected those having HGNC approved gene nomenclature. We mapped each protein to the corresponding gene symbol removing redundant interactions. Finally, we obtained a network with 179,387 interactions and 15,498 gene symbols. Then, we extracted the *induced iRefIndex* network by TCPA gene set such that each node in the network is a gene symbol within TCPA gene set. In the rest of the paper, we simply name it as *iRefIndex* network or gold standard. INBIA gold standard contains 148 nodes and 972 edges.

### Inference network based on iRefIndex analysis-INBIA pipeline

**Fig. 1:**
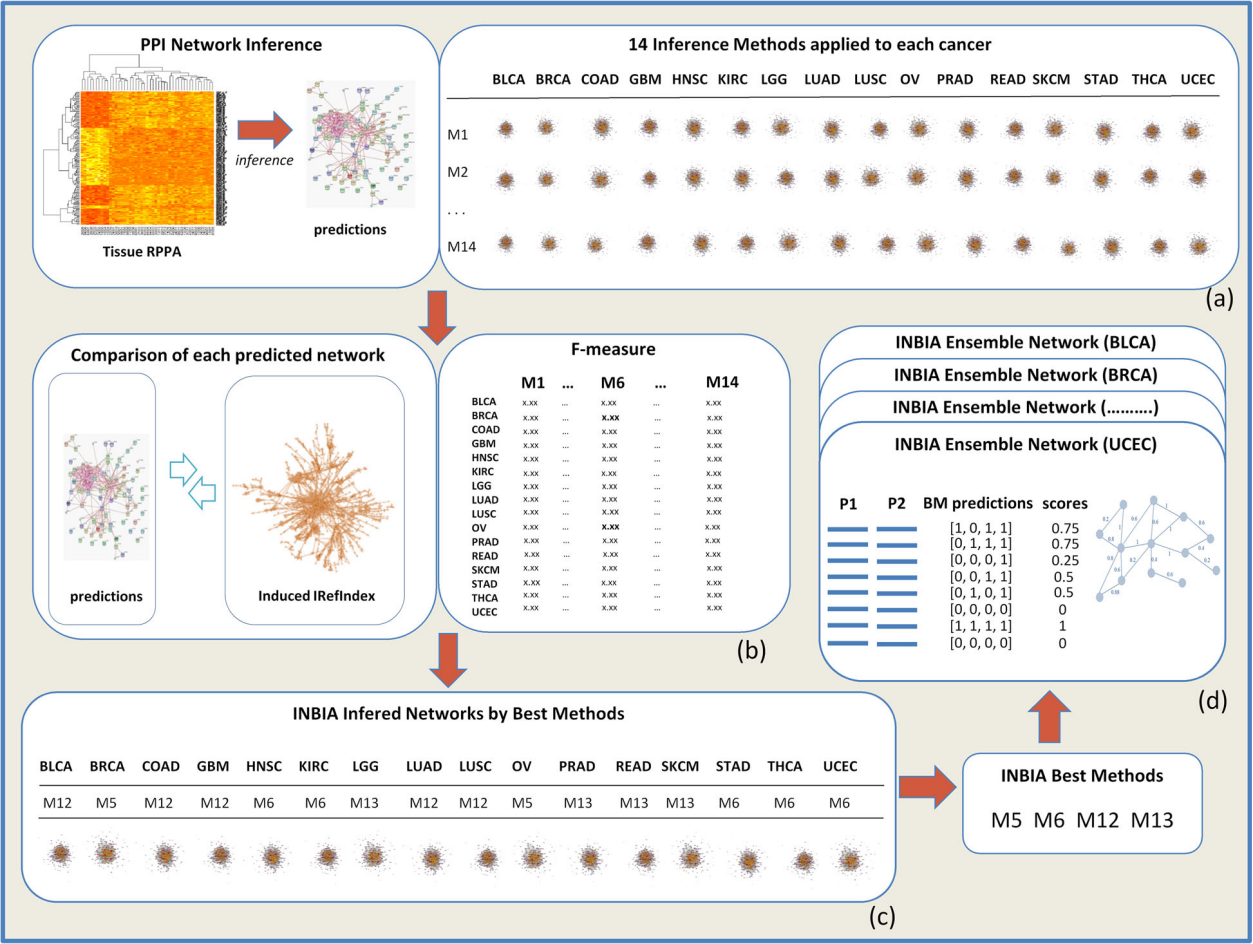
Inference Network Based on iRefIndex Analysis (INBIA) pipeline. We selected 14 inference methods and applied them to the 16 RPPA datasets in order to achieve PPI predictions (**a**). Networks are inferred following two approaches: (i) the predictions have been compared with the gold standard, iRefIndex, in order to obtain true positive (TP), false positive (FP), true negative (TN) and false negative (FN) values from which it was computed F-measure, a weighted combination of precision and recall (**b**). The best method for each cancer type was selected and its associated network was returned (**c**); (ii) for each cancer type, an ensemble network was created by computing all possible PPIs generated from genes associated to TCPA proteins and then a score from the ensemble of best methods (BM) that represents the percentage of BMs within the ensemble which have predicted that PPI (**d**). The methods named M1, M2, …M14 correspond to those reported in Additional file 1:Table S2

INBIA relies on the selection of a subset of best performing methods made through comparisons with the gold standard. Figure 1 depicts INBIA methodology to infer protein network interactions from TCPA proteomic expressions for 16 cancers tissues (details on data are reported in Results and discussion Section). INBIA applies the 14 methods (See Fig. 1(a) and Additional file 1: Table S2), using their default parameters.

Following the Dialogue on Reverse Engineering Assessment and Methods (DREAM) Challenge for gene regulatory network inference [14], we classified the methods into four main classes: *correlation/partial correlation*, *linear regression* and *mutual information*[7] (Table 1). Results have been then filtered according to the significance of the method’s predictions. In particular, for each predicted protein-protein edge a *p*-value, based on Graphical Gaussian Model [15], is computed. For methods based on mutual information we applied their own criterion to filter false positive interactions and maintained only those with scores greater than 0. For all the remaining methods, only predicted interactions with *p*-value less than 0.05 have been selected.

**Table 1:** Inference methods used to retrieve predictions on PPI from cancer tissues grouped by category

Category	Method name
Correlation	Spearman correlation [SPEARMAN]
	Pearson correlation [PEARSON]
	TOM Similarity [WGCNA]
Partial Correlation	Simple partial correlation [SPC]
	GeneNet shrunken [GENENET]
	Graphical lasso [GLASSO]
Regression	Partial least squares regression [PLS]
	Ridge regression [RIDGE]
	Lasso regression [LASSO]
	Elastic net regression [ELASTICNET]
Mutual Information	ARACNE additive [ARACNEA]
	ARACNE multiplicative [ARACNEM]
	Context likelihood of relatedness [CLR]
	Maximum relevance minimum redundancy [MRNET]

For each inferred network, INBIA measures the goodness of predictions starting from the computation of true positive and false positive rates with respect to the gold standard. After the execution of each inference algorithm, INBIA collects the most significant results and measures these values. The True Positives (TP) are all the predicted interactions that are present in the gold standard. True Negatives (TN) are the interactions that are not predicted by the method and are not present in the gold standard. False Positives (FP) are the predicted interactions that are not present in the gold standard. Finally, False Negatives (FN) are the edges in gold standard that are not obtained by the method. The Precision is then defined as the ratio of TP over the sum of TP and FP. Recall is the ratio of TP over the sum of TP and FN. The efficiency of the methods is established by combining precision and recall into the F-measure:

1 $$ {F-measure} = 2 \cdot \frac{precision \cdot recall}{precision + recall}  $$

From each inferred network, we built three more networks in the following way: for each pair of nodes not connected by a direct edge, we computed indirect interactions by searching for paths of length *k* within the gold standard, for *k*=2,3,4, respectively. Such interactions are referred as *indirect*. From now on we will only refer to inferred networks with direct edges (i.e., *k*=1).

For each cancer type, INBIA infers networks by applying the 14 methods and choosing as result the network with higher value of F-measure (Fig. 1(b)(c)). The method which generates such network is named ’best method’. Once the best method has been established for each of the 16 cancer tissues, an ensemble network, for each cancer type, is built by taking into account all networks inferred by the best methods (Fig. 1(d)). INBIA creates complete networks whose nodes are TCPA genes and all possible edges among them exist. Then it assigns scores to edges, initially set to zero. Scores represent how many methods have predicted the edges. At the end, edges with score zero are removed from the network. Unless differently specified, we refer as INBIA inferred networks those obtained by the best methods and not through the ensemble computation.

### INBIA validation

#### Comparison with pathway commons PPI network

The selection of a good gold standard for human PPI network inference affects the quality of the results. We also created a gold standard of protein interactions network from Pathway Commons v2 as presented in [4]. We selected the ontology contained in the file *Pathway Commons all.BIOPAX.owl* and downloaded bp_prior v2.9.1 that implements Prior Extraction and Reduction Algorithm (PERA) (https://bitbucket.org/armish/bp/_prior).

PERA accepts as input a list of proteins together with their post-translational modifications status and a file containing pathways in BioPAX format. We downloaded the input list provided in the supplementary files of [4]. Such list contains only TCPA proteins with related official gene symbols and associated PTMs. PERA produced as output a list of direct or indirect (with mediating proteins) PPIs found within the pathways relative to genes provided in input. The parameters –graph-limit and –site-tolerance were set to 1 and 3, respectively, for direct interactions. Proteins were converted with official gene symbols achieving 790 unique interactions (See Additional file 2). We ran the INBIA pipeline by using as gold standard the Pathway Commons resource as described above. We then compared the results with the original INBIA pipeline which uses iRefIndex. For sake of simplicity, we simply refer to the pipeline based on Pathway Commons as PERA.

#### Topology-based analysis

Inferred networks were compared with online resources in order to assess their quality in terms of corrected tissue specific predicted interactions. Negatome 2.0 [8] reports likely non interacting proteins based on literature mining and protein structures from Protein Data Bank (PDB)[16]. This dataset is useful to evaluate the number of false positives in predicting the PPIs. Negatome distinguishes between PDB and manually derived data or combines them in order to create a unique resource. We chose the latest stringent solution containing 6,136 PPIs with UniProt symbols subsequently converted in a total of 5,386 likely non-interacting proteins with official gene symbols.

TissueNet v.2 [9] and GIANT [10] contain functional interaction networks for several human tissues. In particular INBIA uses TissueNet, a database of tissue specific networks obtained from HPA protein expression (Human Protein Atlas). GIANT is based on a Bayesian methodology to integrate data from genome experiments and disease conditions. GIANT classifies, for each tissue, the edges into four classes C1-C4. C1 contains interactions among tissue specific genes positively co-expressed in the tissues, C2 contains interactions among tissue specific genes and multi-tissues genes positively co-expressed. C3 and C4 are the negative-co-expressed counterparts of C1 and C2, respectively. Validation was carried out by choosing key-words such that cancer tissues matched normal counterparts. Trustfulness on edges prediction relies on the edges’s presence in TissueNet, in C1 and C2 GIANT classes, and their absence in Negatome. In order to evaluate the reliability of INBIA in predicting tissue specific networks from new pathological conditions, by using the ensemble networks and tissue specific PPI networks from TissueNet, we computed precision-recall (PR) curves with the R package ROCR [17]. We performed a topological analysis of the INBIA and PERA inferred networks, by making a functional annotation of their nodes and by looking for small network motifs in the annotated networks. A network motif is a subgraph of the target network which occurs more frequently than expected with respect to a random network model [14, 18, 19]. The topological information of motifs may provide insights on the main processes involving specific tissues and may help to identify the main active pathways. Moreover, predicted PPIs within recurrent motifs may be present or not across the inference methods, thus being putative biomarkers to look at. We ran FlashMotif algorithm [20] to find all possible non-induced colored motifs of 3 and 4 nodes, where a motif represents a subgraph in which each node is ‘colored‘ with a specific GO term. We used BiNGO [21] to annotate nodes of INBIA and PERA inferred networks with one or more of the following Gene Ontology terms: phosphorylation (GO:0016310), cell death (GO:0008219), signaling (GO:0023052) and cell proliferation (GO:0008283). Proteins linked to none of the previous GO terms were annotated with the generic ‘biological process‘ GO term (GO:0007582). We decided to focus only on non-induced motifs because the number of non-induced occurrences of a motif is less sensitive to the presence of false positive interactions in a network, so the non-induced definition is more suitable than the induced one for PPI networks [22]. Since a node can be associated with two or more GO terms and FlashMotif only works with graphs where each node is mapped with a unique color, we therefore re-structured the networks, before running FlashMotif. In particular, if a node had *n* associated GO terms we created *n* copies of the node together with *n* copies of its links to the other nodes in the network. Replicating nodes and edges is necessary to avoid loosing network information and underestimating the number of occurrences of labeled motifs.

#### Functional analysis

The functional enrichment of predicted interactions was carried out using Molecular Signature Database v5.2 (MSigDB) for gene set enrichment analysis [23]. Overlaps with MSigDB gene sets were computed by selecting the top 10 with false discovery rate less than 0.05 within hallmark gene sets which yield precise biological functions relationships and contain genes with similar expression patterns.

The gene mutation analysis of interacting proteins was performed using Catalogue Of Somatic Mutations In Cancer (COSMIC) v80 [24], in particular we used the information contained in the cancer gene census. For each cancer type, the proteins contained in the PPI networks were extracted and annotated with COSMIC data considering somatic and germline mutations.

## Results and discussion

We analyzed the performance of INBIA and PERA by measuring the biological soundness of the inferred networks together with their functional annotation with cancers. Additional file 1: Table S3, summarizes the topological properties of the inferred networks while the measures are reported in Additional file 3. We computed Wilcoxon rank-sum test, using the package stats in R, in order to verify if the networks produced by INBIA and PERA were significantly different based on their network properties (See Additional file 1: Table S3, along with *p*-values produced by the statistical test). As the *p*-values were never less than 0.05, we could not rejected the hypothesis, therefore, we concluded that networks were comparable. Intuitively, the difference in the number of interactions is due to the nature of the resources used by iRefIndex and Pathway Commons and their curation. iRefIndex is made of a large number of interactions. However, this may be a drawback since it could increase the number of false positive in the networks inferred for each cancer type. We will verify this aspect, i.e. the accuracy of obtained computational protein interactions, by using further validation procedures.

**Fig. 2:**
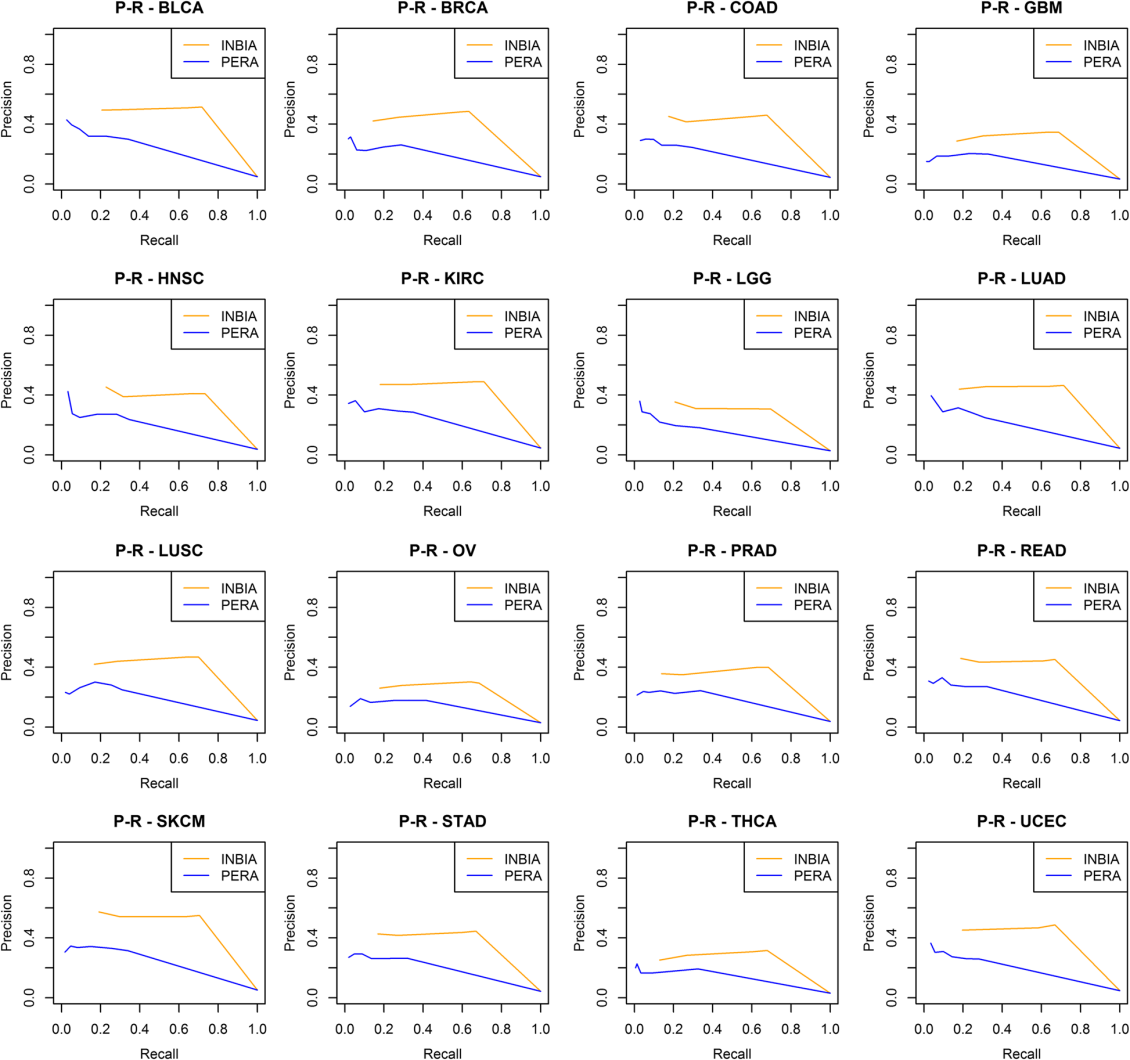
Network prediction quality based on tissue specificity. Precision-recall curves of INBIA’s (orange line) and PERA’s performances (blue line) in predicting tissue-specific PPIs. Each plot refers to a specific cancer type. The performances were computed by considering the ensemble scores generated from INBIA’s and PERA’s best methods and the TissueNet counterparts as ground truth (see Additional file 1: Table S5)

Table 2 reports the F-measures of the best methods of INBIA and PERA. Additional file 1: Table S4, reports also the F-measures for indirect interactions. From the best methods, we computed tissue-specific precision and recall (PR) curves by using the ensemble inference networks for both approaches (Fig. 2). INBIA’s ensemble set is made by 4 methods (CLR, GLASSO, PLS, and MRNET), while PERA’s best methods are: ELASTICNET, PLS, ARACNE, SPEARMAN, WGCNA, and CLR. Tissue specific networks from TissueNet are used as gold standards to compute PR curves. PR curves assess the goodness of INBIA and PERA in inferring pathological networks starting from the ensemble scores assigned to PPIs. For all cancers, INBIA performs better than PERA even if, for some tissues, the difference between the curves is reduced. INBIA’s precision is constant at 0.4 on average until recall reaches 0.7, then it decreases to a value less than 0.1 when recall is maximum. It achieves the best results for BLCA and SKCM. This trend can be associate to a PR curve of a good classifier meaning that the predictions of INBIA based on iRefIndex are more accurate compared to those of PERA based on Pathway Commons.

**Table 2:** Cancer types and best performing inference network methods with maximum F-measure value

Cancer Type	INBIA	PERA
BLCA	CLR (0.188)	ELASTICNET (0.179)
BRCA	GLASSO (0.186)	PLS (0.179)
COAD	CLR (0.182)	ARACNE (0.166)
GBM	PLS (0.196)	PLS (0.191)
HNSC	PLS (0.184)	PLS (0.178)
KIRC	PLS (0.210)	PLS (0.180)
LGG	PLS (0.193)	PLS (0.194)
LUAD	CLR (0.187)	SPEARMAN (0.188)
LUSC	CLR (0.184)	SPEARMAN (0.184)
OV	GLASSO (0.191)	PLS (0.174)
PRAD	MRNET (0.191)	WGCNA (0.168)
READ	MRNET (0.186)	CLR (0.166)
SKCM	MRNET (0.188)	WGCNA (0.166)
STAD	PLS (0.179)	PLS (0.165)
THCA	PLS (0.196)	PLS (0.174)
UCEC	PLS (0.189)	WGCNA (0.178)

Networks obtained from the best methods for both PERA and INBIA were compared with two datasets in order to assess their quality in terms of corrected predicted interactions (Table 3). We associates TCPA cancer tissues and related genes to normal counterparts, from online resources, by considering the incidence of each pathology in normal tissues (See Additional file 1: Table S5). Comparing with Negatome, we found that there was, in all cases and for both methods, a very small set of interactions in common, meaning that both methods predicted few validated false negative interactions. However, the percentage of overlapping interactions of our method is lower compared to that of PERA. As reported in Table 3 and Additional file 1: Table S6, compared to PERA, INBIA predicts a larger statistically significant amount of tissue specific protein interactions present in TissueNet (*p*-value <0.001 by using T-test).

**Table 3:** Comparisons with Negatome and TissueNet by considering cancer types and normal counterparts (see Additional file 1: Table S5). The overlap is reported in percentage with respect to the number of total unique predictions per cancer/tissue. For each tissue, we report in the first row the percentage of INBIA’s overlapping, while in the second PERA’s one

Cancer type	Negatome (%)	TissueNet (%)
BLCA	*0.745*	51.565
	1.463	44.390
BRCA	*0.247*	47.654
	0.830	29.461
COAD	*0.609*	44.901
	0.806	34.274
		30.323
GBM	*0*	36.452
		28.064
		25.484
		19.636
	0.727	26.909
		20.727
		15.636
HNSC	*0.69*9	43.706
	0.820	31.967
KIRC	*1.608*	48.231
	2.137	40.171
LGG	*0.62*	32.812
	1.020	25.510
LUAD	*0.711*	46.373
	2.367	41.420
LUSC	*0.895*	46.418
	3.297	43.407
OV	*0.665*	25.942
	1.060	22.261
PRAD	*0.602*	39.458
	0.395	32.411
READ	*1.064*	43.769
	1.431	38.998
SKCM	*0.446*	54.018
	0.704	42.958
STAD	*0.315*	43.218
	0.772	35.135
THCA	*0.308*	26.769
		40.308
	0.763	19.466
		26.336
UCEC	*0.615*	48.615
	1.463	37.073

Text highlighted in italic refers to our method (INBIA), the second one to PERA

**Fig. 3:**
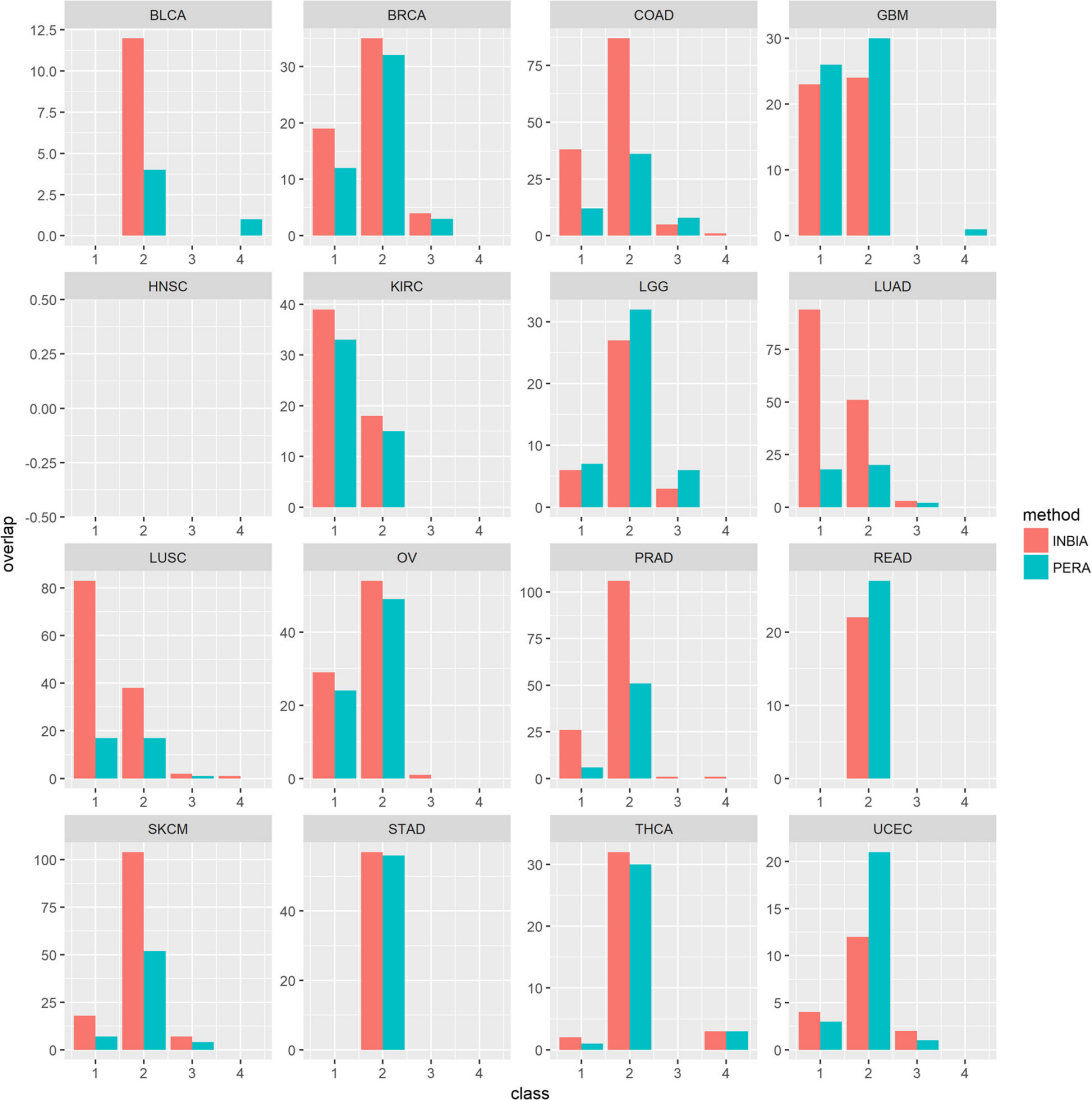
Comparison of predicted PPI classes in GIANT. The plots show comparison between INBIA and PERA best methods for each cancer type and relative amount of predicted PPIs that fall within each GIANT class. PPIs in C1-C2 represent functionally related pairs in the same tissue (C1) or in multi-tissues (C2), conversely C3-C4 are likely functionally unrelated pairs. For HNSC no overlaps were found, meaning that both methods cannot predict the interactions provided by GIANT

Figure 3 reports the PPI predictions present in tissue specific networks from GIANT. INBIA and PERA maintain the same trend: the majority of predictions belong to classes C1 and C2, with some exceptions that show small amounts of PPI in C3, and C4 classes. Once again, the amount of predictions predicted of INBIA is larger than PERA but the differences in this case has a weak statistical significance (*p*-value = 0.06 by using T-test). Concerning motif analysis, FlashMotif took about 4 minutes to retrieve all colored motifs with 3 and 4 nodes in all the 16 INBIA and PERA tissue-specific networks. The algorithm found 959 colored motifs with 3 nodes and 9,006 motifs with 4 nodes in INBIA networks. In PERA networks, FlashMotif found 798 motifs with 3 nodes and 5,489 motifs with 4 nodes. However, very few of them were significantly over-represented (*p*-value ≤ 0.05). FlashMotif found just 7 over-represented motifs with 4 nodes in the INBIA network. In PERA network, 38 motifs with 3 nodes and 903 motifs with 4 nodes were over-represented. The higher number of over-represented motifs found in PERA networks is mainly due to the fact that these networks are sparser than INBIA ones. Notwithstanding, interestingly, all 7 over-represented motifs with 4 nodes found in INBIA networks are also over-represented motifs in PERA networks. Table 4 lists these motifs and, for each motif, it reports the tissue where the motif is over-represented, the number of occurrences, and relative *p*-values. The 7 over-represented motifs found are examples of ‘diamonds’, which are common in signal-transduction networks, because they are related to modifications such as phosphorylation [25]. So, motif analysis shows that protein-protein interactions of networks inferred by INBIA are biologically significant. We computed the predicted PPIs in common among all inferred networks (See Additional file 1: Figure S1). Each node size in the INBIA’s and PERA’s network corresponds to collective influence and nodes are colored in red if they belong to MDS. INBIA graph has one large connected component. The graph produced for PERA has 5 small components. According to INBIA results we can speculate that a core module may be preserved in different cancers. The list of all edges found with this analysis is reported in Additional file 4. We investigated the role of genes in the inferred networks by considering the Oncopanel, a custom targeted next-generation sequencing assay for cancer [26]. We found a core module containing 15 genes that overlap with OncoPanel (See Additional file 1: Figure S2). Moreover, for each cancer type, the best PPI inference networks related to OV, BRCA, UCEC, STAD and KIRC achieved the highest number of genes (33, 28, 27, 26, 24, respectively) that overlap with OncoPanel. The complete results are reported in Additional file 5. Minimum dominating sets (MDS) [27] and collective influence of nodes (See Additional file 1: Section 3 for the definition) for each inferred network by INBIA and PERA are reported in Additional file 6. INBIA unravels a total of 83 PPIs that includes EIF4EBP1, AKT1, GSK3A, RPS6, MAPK1, SRC proteins as most central in the network based on computed collective influence centrality measure [28]. PERA achieves 12 common PPIs in which the main central nodes are TSC2, AKT1 and GSK3A.

**Table 4:** Common over-represented motifs found in INBIA and PERA networks inferred from best methods. For each motif, the number of occurrences and relative *p*-values in INBIA and PERA networks are reported (CD = Cell Death, CP = Cell Proliferation, P = Phosphorylation, S = Signaling)

Motif	Cancer type	INBIA	PERA
	THCA	6256 (0.034)	7 (2.66E-04
	LGG	10477 (0.039)	30 (3.86E-06)
	THCA	4230 (0.042)	3 (0.001)
	LGG	6086 (0.047)	24 (2.60E-06)
	OV	4159 (0.048)	60 (3.28E-06)
	THCA	2898 (0.047)	2 (0.003051681)
	THCA	7039 (0.038)	7 (1.79E-05)

**Fig. 4:**
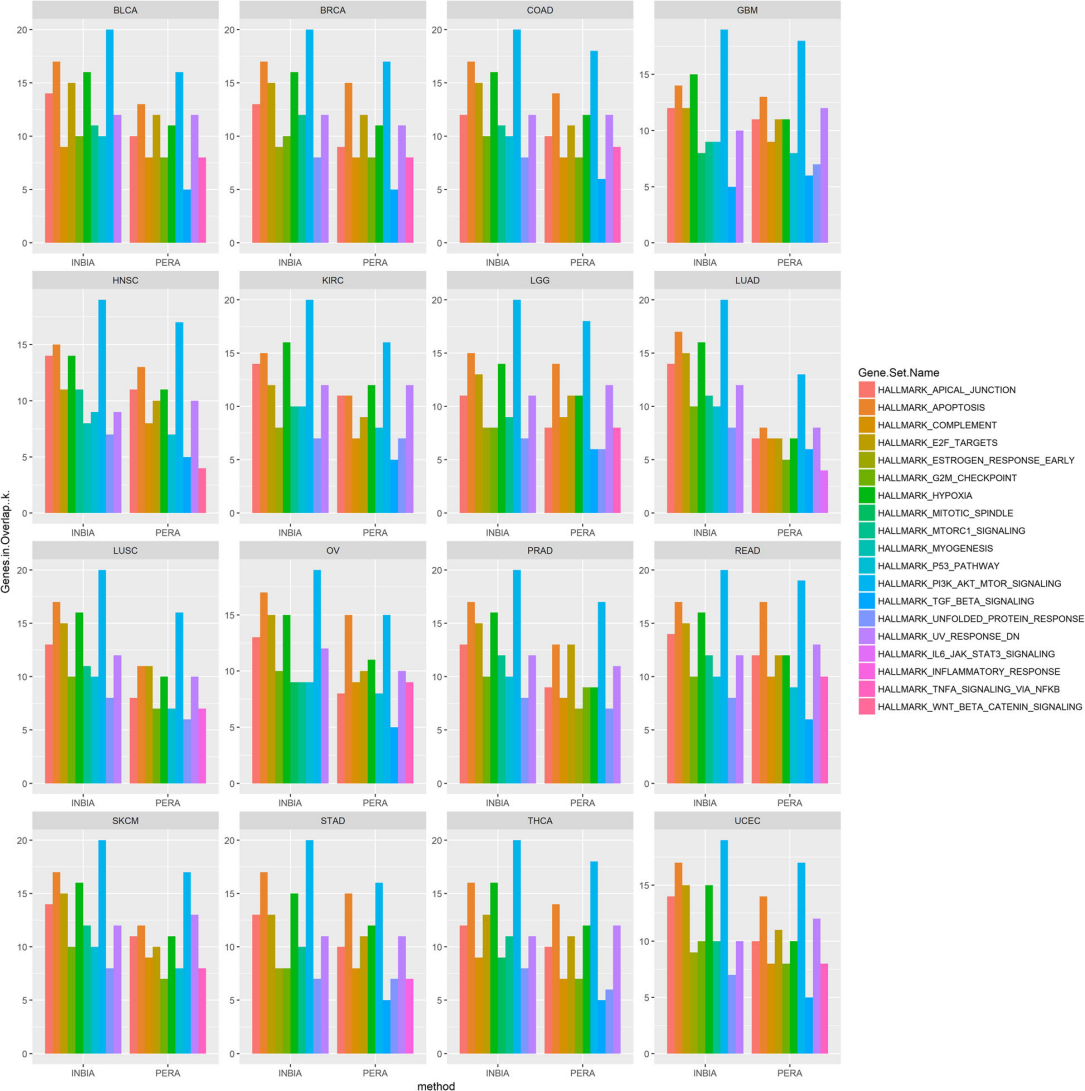
Gene Set enrichment analysis. The plot represents the most enriched gene sets for each cancer type. For each type, the best methods predictions based on *INBIA* and *PERA* were retrieved and the gene symbols corresponding to the predicted PPIs were enriched by using MSigDB. The x-axis contains the hallmark gene sets for each method while y-axis reports the gene symbols *k* that overlaps with hallmark gene sets, reported in the legend with different colors

Additional file 1: Table S7, reports the enrichment of the genes present in the whole TCPA dataset. Figure 4 shows the enriched gene sets from MSigDB [23] for INBIA and PERA. The analysis was carried out by using PPIs predicted from best methods for each cancer type, as reported in Table 2. Note that each network does not contain all TCPA proteins, since only nodes with predicted interactions belong to the network. The x-axis contains the number of genes overlapping with each gene set while the y-axis represents the enriched gene sets. HALLMARK_PI3K_AKT_MTOR_SIGNALING always contains the majority of genes for all tissues both for INBIA and PERA. The latter in general predicts less PPIs and this can be seen in the smaller number of overlapping genes. INBIA’s best networks produce specific gene sets, e.g. HALLMARK_MTORC1_SIGNALING, that are not present in PERA’s results. Conversely, HALLMARK_INFLAMMATORY_RESPONSE or HALLMARK_TNFA_SIGNALING_VIA_NFKB are not among gene sets enriched from INBIA.

Table 5 reports the genes inferred by INBIA and PERA annotated in COSMIC. Once again, our method, in general, infers more mutated genes associated with specific cancers.

**Table 5:** COSMIC mutation analysis by considering genes related to PPIs predicted from best methods in our and PERA approaches and the relative amount of somatic and germline mutated genes

Cancer type	Somatic mutation	Germline mutation
BLCA	ERBB3,NOTCH1,TSC1	–
BRCA	*AKT1,ARID1A,BAP1,BRCA2,CCND1,CDH1,CDKN1B*,	
	*ERBB2,ESR1,NOTCH1,PIK3CA,RB1,TP53*	*BRCA2,CHEK2,RB1,TP53*
	AKT1,BRCA2,CCND1,CDH1,CDKN1B,ERBB2,ESR1,	BRCA2,CHEK2,TP53
	GATA3,NOTCH1,PIK3CA,TP53	
COAD	ERBB3	–
GBM	PIK3CA,PIK3R1	–
HNSC	ERBB3,MTOR,NOTCH1,TSC2	–
KIRC	*MET,MTOR,NF2,TSC1,TSC2,VHL*	*MET,TSC1,TSC2,VHL*
	MET,MTOR,TSC1,TSC2,VHL	
LGG	BRAF,EGFR,PTEN,RAF1,TP53	ATM,PTEN,TP53
LUAD	*NOTCH1,RB1,TP53*	*RB1*
	–	–
LUSC	*NOTCH1,RB1,TP53*	*RB1*
	TP53	–
OV	*AKT1,ARID1A,BRAF,BRCA2,CCNE1,CTNNB1,ERBB2,*	*BRCA2,MSH2,MSH6,STK11*
	*MAPK1,MSH2,PIK3R1*	
	AKT1,BRAF,CCNE1,CTNNB1,ERBB2,MAPK1,PIK3R1	–
PRAD	*AR,BRAF,NDRG1,PTEN,RAF1*	*PTEN*
	AR,BRAF,RAF1	–
READ	*AKT1,BRAF,CTNNB1,MAP2K1,MSH2,MSH6,PIK3CA,*	*MSH2,MSH6*
	*PIK3R1,SMAD3,SMAD4,SRC,TP53*	
	AKT1,BRAF,CTNNB1,MAP2K1,PIK3CA,PIK3R1,SMAD3,SMAD4,SRC,TP53	–
SKCM	ERBB3,NOTCH1	–
STAD	BRAF,CDH1,ERBB2,ERBB3,PIK3CA	CDH1
THCA	*BRAF,MTOR*	*CDKN1B*
	BRAF,NRAS	
UCEC	*MSH2,MTOR,PTEN,SRC*	*MSH2,MSH6,PTEN*
	MTOR,PTEN,SRC,YWHAE	PTEN

For each cancer type, tuple highlighted in italic refers to our method (INBIA), the second one to PERA. When only one record per cancer type is present then it means that the two approaches achieved the same results

## Conclusions

In this paper we aimed to understand and therefore overcome some of the limitations affecting the performances of network inference methods. We proposed a methodology for protein-protein interaction network prediction from proteomic data, called Inference Network Based on iRefIndex Analysis (INBIA). INBIA consists of several inference methods based on correlation, partial correlation, mutual information and regression. It uses as gold-standard protein interaction networks (iRefIndex). The reliability of our approach is assessed on a set of tissue-specific PPI networks built on top of TCPA data. We compared INBIA with PERA [4], which instead relies on Pathway Common database as gold-standard. The results show that our approach is capable to recover better PPI interaction networks, in terms of precision-recall, with respect to those retrieved by PERA when compared on TissueNet [9]. Furthermore, a comparison of the inferred networks using GIANT tissue/edges classification shows that INBIA networks contain more interactions among tissue specific genes positively co-expressed in the tissue. Moreover, INBIA retrieves interactions involving a larger set of known cancer mutated genes and networks contain significantly expressed motifs. INBIA results show a consistent core module of interactions preserved in different cancers, composed of topological significant nodes, i.e. with high collective influence and belonging to minimum dominating sets. On the other hand both INBIA and PERA return very few false positive interactions with respect to the Negatome. Therefore, the comparison clearly highlights that the selection of a proper reference database is crucial to establish the actual soundness of inference network models.

## Additional files

Additional file 1 Supplementary Materials. (PDF 1120 kb)

Additional file 2 PERA PPI gold standard network created from Pathway Commons v2. (XLSX 19 kb)

Additional file 3 Inferred PPIs network statistics and *p*-values computed with Wilcoxon rank-sum test for each cancer type. (XLSX 14.9 kb)

Additional file 4 Methods and cancers consensus PPI networks for INBIA and PERA. (XLSX 15.4 kb)

Additional file 5 Genes present in OncoPanel that overlap with TCPA, INBIA consensus network and INBIA cancer-specific best PPI networks. (XLSX 18.3 kb)

Additional file 6 Minimum dominating set and corresponding collective influence measure as computed within each cancer’s best method network for INBIA and PERA. (XLSX 49 kb)

## Abbreviations

DPI: Data processing inequality
PPI: Protein-protein interaction
PTM: Post-translational modification
RPPA: Reverse-phase protein array
