# Supplementary material for: INBIA: a boosting methodology for proteomic network inference
Source: BMC Bioinformatics. 2018 Jul 9;19(Suppl 7):188. doi: 10.1186/s12859-018-2183-5 (PMC6069689; doi:10.1186/s12859-018-2183-5)
Supplement: Supplementary file 1 — Supplementary Materials. (PDF 1120 kb) [file 12859_2018_2183_MOESM1_ESM.pdf]

RESEARCH

Supplementary File INBIA: a boosting methodology for proteomic network inference

Davide S Sardina[1], Giovanni Micale[1], Alfredo Ferro[2], Alfredo Pulvirenti[2] and Rosalba Giugno[3*]

*Correspondence: rosalba.giugno@univr.it
[3] Department of Computer Science, University of Verona, Strada le Grazie 15, 37134 Verona, Italy
Full list of author information is available at the end of the article

1 Methods and parameters

We selected 14 network inference methods based on several statistical measures, such as: correlation, mutual information, entropy maximization, regression. The methods were applied to reverse-phase protein arrays (RPPA) datasets retrieved from TCPA containing expression levels of 190 proteins and phosphoproteins in 16 cancer types (Table S1). Below we report the description of the procedures used to execute the methods. The complete analysis was implemented in R language. Table S2 reports the used R packages and parameters.

Network inference methods description

Concerning correlation, we used *Pearson*, *Spearman*, and *Partial Correlation*. The last measure has been computed using the inverse of the Pearson correlation matrix through the *R* function *cor2pcor* which makes use of Singular Value Decomposition (SVD) [1]. Among correlation-based approaches, the *Weighted correlation network analysis (WGCNA)* yields module detection, network of co-expressed genes, gene significance evaluation and topological analysis [2]. We used the Topological Overlap Matrix (TOM) similarity [3] to predict putative edges between proteins in the datasets. The analysis have been carried out by considering the best power computed with `pickSoftThreshold` for correlation network reconstruction such that it resembles a scale-free topology. The `blockwiseModules` function reconstructs the network from data and finds module of interconnected nodes with minimum module size of 30. The dynamic tree cut approach to select best cut was also assessed with `cutreeDynamic` function by using average linkage hierarchical clustering of dissimilarity TOM matrix $(1 - TOM)$.

The Ridge and Lasso regression methods compute penalized estimates of the regression coefficients which tend to vanish when using $L2$ or $L1$ norm, respectively. These methods are applied to the inverse covariance matrix. Ridge method computed from `ridge.net` creates a vector *lambda* of $1,000$ penalty terms that depends on the number of samples n and the number of splits in k-fold cross-validation, then applies ridge regression.

The advantage of Lasso regression represents its low computational complexity. For this reason, it has been applied to high dimensional data [4]. On the other hand the main shortcoming to work on biological data is that it needs space reduction procedures to reduce false positives. The implementation through `adalasso.net` yields best coefficient from cross-validation-optimal regression, then computes partial correlation.

Table S1 Cancer types from TCPA analyzed in this work and related number of samples.

Cancer Type	Abbreviation	Observations
Bladder Urothelial Carcinoma	BLCA	127
Breast invasive carcinoma	BRCA	750
Colon adenocarcinoma	COAD	334
Glioblastoma multiforme	GBM	215
Head and Neck squamous cell carcinoma	HNSC	212
Kidney renal clear cell carcinoma	KIRC	454
Lower grade glioma	LGG	260
Lung adenocarcinoma	LUAD	237
Lung squamous cell carcinoma	LUSC	195
Ovarian serous cystadenocarcinoma	OV	412
Prostate adenocarcinoma	PRAD	164
Rectum adenocarcinoma	READ	130
Skin Cutaneous Melanoma	SKCM	207
Stomach adenocarcinoma	STAD	299
Thyroid carcinoma	THCA	375
Uterine Corpus Endometrial Carcinoma	UCEC	404

Table S2 Methods, functions and default parameters used in the analysis.

ID	Method name	Command	Parameters	Library
M1	PEARSON	`cor`	`method = "pearson"`	stats
M2	SPEARMAN	`cor`	`method = "spearman"`	stats
M3	SPC	`cor2pcor`	`cor(pearson)`	corpcor
M4	GENENET	`ggm.estimate.pcor`		GeneNet
M5	GLASSO	`glasso`<br>`invcov2parcor`	`cov(data), rho=0.01`<br>`glasso$wi`	glasso
M6	PLS	`pls.net`		parcor
M7	RIDGENET	`ridge.net`		parcor
M8	LASSO	`adalasso.net`		parcor
M9	ELASTICNET	`elasticNetwork`	`nfold = 3, alpha = seq(0.01, 0.99, length.out=10)`	
M10	ARACNEA	`aracne.a`	`mi = knnmi.all(data)`	parmigene
M11	ARACNEM	`aracne.m`	`mi = knnmi.all(data)`	parmigene
M12	CLR	`clr`	`mi = knnmi.all(data)`	parmigene
M13	MRNET	`mrnet`	`mi = knnmi.all(data)`	parmigene
M14	WGCNA	`TOMsimilarityFromExpr`	`networkType = "unsigned",`<br>`TOMType = "unsigned",`<br>`power = best.power`	WGCNA

Graphical lasso solves the lack of reductions procedures by using the block coordinate descent algorithm. Elastic Net combines the Lasso and Ridge penalties achieving better results compared to Lasso with several real world data [5]. Complementary approaches, e.g. *Shrinkage (Gene net)* and *Partial Least Square Regression*, try to estimate covariance matrices in order to infer dependencies among genes. Results were filtered according to the significance of the method's predictions. In particular, for each predicted protein-protein edge a p-value, based on Graphical Gaussian Model [1], is computed. Graphical Gaussian models are used to describe gene association networks from partial correlation. The original model hypothesizes that the genomic data follows a multivariate normal distribution and has positive definite covariance matrix. It states that correlation coefficients represent direct or undirect regulations by common genes. The higher the partial correlation between two genes is the more is the strength of the direct interaction between them. It further computes the partial correlation matrix from the inversion of the correlation coefficients. This model has been improved for small sample size in [1] by estimating partial correlation coefficients from the inversion of the correlation matrix with Moore–Penrose pseudoinverse matrix and then used bootstrap aggregation to stabilize the estimator.

ARACNE [6], *CLR* [7], and *MRNET* [8] are based on the estimation of the mutual information between variables. *ARACNE* exploits the *data processing inequality*

(DPI) to delete false positives (i.e. indirect edges). It has been applied both on synthetic and biological gene expression data to create large networks with more than $120,000$ gene interactions. *CLR* computes the distribution of the values of mutual information for all the pairs of variables. Next, it applies the relevance network approach to filter non-significant interactions. *Minimum-redundancy-maximum-relevance (MRNET)* algorithm selects the variables with maximum relevance in terms of mutual information. The indirect interactions caused by interdependence among selected variables are penalized through a minimum redundancy criterion.

2 Inferred Networks Properties and Statistics

Table S3 reports the topological measures in the networks inferred by our approach INBIA with respect to those computed by PERA [9]. For each undirected network we computed:

1 Number of nodes;
2 Number of edges;
3 Edge density: the ratio of the network edges and the total possible edges;
4 Transitivity: the global transitivity represents the number of triangles divided by the number of triples in the network;
5 Diameter: the maximum length of the shortest path between any two vertices in the network;
6 Degree centralization: obtained from vertices degree, represents the level of network centralization normalized by the maximum centrality level;
7 Closeness centrality: similar to degree centralization, is the normalized closeness centralization index, where the closeness for each vertex is defined as $C(v) = \frac{1}{\sum_u d(u,v)}$, where $d(u,v)$ is the shortest path connecting nodes u and v in the network;
8 Betweenness centrality: for each vertex v, the fraction of shortest paths between every other two nodes in the networks that contain v as an intermediate vertex;
9 Mean distance: the average of the length of all network's shortest paths.

Table S4 reports the F-measure, i.e. a combination of precision and recall, considering *direct* ($k = 1$) and *indirect* ($k = 2, 3, 4$) interactions, where k represents the length of paths within the gold standard connecting the proteins. For each tissue, the best method is selected by computing the maximum F-measure across all methods.

To assess the quality of predicted edges, inferred networks are compared with tissue-specific networks from TissueNet v.2 [10] and GIANT[11]. For example, in order to find the best match between cancer and normal counterpart we sought if a specific cancer can occur in that tissue, e.g. BLCA corresponds to urinary_bladder, BRCA to mammary_gland, GBM to brain, PRAD to prostate_gland, and so on. Every file, e.g. urinary_bladder in GIANT, contains in each row an edge between two proteins and the corresponding class as defined in [11]. We verified the overlap of predicted PPIs from INBIA with those contained in the normal counterpart in GIANT and collected the classes. Cancer types were associated to tissues in TissueNet v.2 and GIANT following the search keywords reported in Table S5.

Table S3 Properties of inferred networks: Number of nodes and edges(N, E), Edge density (ED), Transitivity (T), Diameter (D), Degree centrality (DC), Closeness centrality (CC), Betweenness centrality (BC), and Mean distance (MD). For each cancer type, the first tuple highlighted in boldface refers to the inferred network from the induced iRefIndex human networks (INBIA) with $k = 1$. The second tuple refers to the inferred networks from Pathway Commons by using PERA, with $k = 1$. The p-value is obtained with Wilcoxon-Rank Test using those measures as components.

Cancer Type	N	E	ED	T	D	DC	CC	BC	MD	pvalue
BLCA	**142**	**671**	**0.06702627**	**0.1963033**	**5**	**0.2166617**	**0.2864414**	**0.1016215**	**2.508341**	**0.4258**
	97	205	0.04402921	0.1918482	8	0.1538875	0.09021319	0.1223768	3.518809	
BRCA	**132**	**405**	**0.04684247**	**0.1471906**	**6**	**0.2432339**	**0.3460722**	**0.1919149**	**2.743697**	**0.8203**
	99	241	0.04968048	0.2042827	9	0.2258297	0.07253683	0.1967823	3.154284	
COAD	**142**	**657**	**0.06562781**	**0.199939**	**5**	**0.2464289**	**0.3044054**	**0.1292083**	**2.521327**	**0.7344**
	103	248	0.04721112	0.1459378	7	0.2174948	0.3073537	0.238328	3.137636	
GBM	**115**	**310**	**0.04729214**	**0.1563299**	**6**	**0.2246377**	**0.107646**	**0.2157567**	**2.844999**	**0.1834**
	105	275	0.0503663	0.2058252	6	0.2669414	0.02724174	0.1849262	2.777559	
HNSC	**114**	**286**	**0.04440304**	**0.1568483**	**6**	**0.2122341**	**0.09678212**	**0.1917622**	**2.908155**	**0.7263**
	94	244	0.05582247	0.1914406	6	0.342027	0.1139639	0.2582048	2.761166	
KIRC	**121**	**311**	**0.04283747**	**0.150108**	**6**	**0.1738292**	**0.09916003**	**0.1354939**	**3.012674**	**0.7263**
	96	234	0.05131579	0.1680672	7	0.2539474	0.1111332	0.2441754	2.927951	
LGG	**114**	**320**	**0.04968173**	**0.1638697**	**6**	**0.215805**	**0.1017394**	**0.220757**	**2.824513**	**0.3627**
	104	294	0.05489171	0.2197422	6	0.2363704	0.04431486	0.1857532	2.833162	
LUAD	**142**	**703**	**0.07022275**	**0.2004164**	**5**	**0.2489262**	**0.3106102**	**0.1096263**	**2.470582**	**1**
	74	169	0.06256942	0.300216	7	0.2798963	0.07108361	0.3380722	3.161357	
LUSC	**144**	**670**	**0.06507382**	**0.2101576**	**5**	**0.2496115**	**0.3134406**	**0.1073905**	**2.546134**	**0.9102**
	87	182	0.04865009	0.3047493	8	0.2536755	0.02952232	0.2165	3.22825	
OV	**132**	**451**	**0.05216285**	**0.1501694**	**7**	**0.2150127**	**0.2993841**	**0.1249435**	**2.719176**	**1**
	101	283	0.0560396	0.1852363	9	0.2739604	0.1075113	0.2447867	3.023908	
PRAD	**144**	**664**	**0.06449106**	**0.197111**	**6**	**0.2362082**	**0.2955189**	**0.1151613**	**2.558858**	**0.7344**
	103	253	0.04816295	0.1758865	8	0.1969351	0.2978846	0.2148186	3.211117	
READ	**143**	**658**	**0.06480843**	**0.182904**	**5**	**0.2450507**	**0.2976828**	**0.113238**	**2.529597**	**1**
	123	559	0.07450353	0.2586023	6	0.2451686	0.09234062	0.1289971	2.610384	
SKCM	**144**	**672**	**0.06526807**	**0.1901693**	**6**	**0.2494172**	**0.3271877**	**0.1368997**	**2.501457**	**0.7344**
	102	284	0.05513493	0.237699	7	0.2121918	0.09705498	0.170387	2.929711	
STAD	**119**	**317**	**0.04515026**	**0.1358713**	**6**	**0.209087**	**0.3025159**	**0.1756729**	**2.941461**	**0.5703**
	102	259	0.0502815	0.1824548	9	0.2467482	0.3245042	0.3063334	3.174723	
THCA	**122**	**325**	**0.04403197**	**0.1443651**	**7**	**0.1956374**	**0.2922507**	**0.1641396**	**2.951633**	**1**
	100	262	0.05292929	0.1897757	8	0.260202	0.1019998	0.2305338	2.971603	
UCEC	**120**	**325**	**0.04551821**	**0.1369458**	**6**	**0.2401961**	**0.3300713**	**0.232514**	**2.886975**	**0.8203**
	96	205	0.04495614	0.1850467	9	0.2287281	0.1029186	0.2576515	3.320448	

Table S6 reports the number of interactions predicted by the compared methods (INBIA and PERA) and the number of such intersections present in TissueNet. The differences of overlaps among INBIA and PERA was statistically significant with p-value < 0.001 (by using T-test).

The Figure S1 reports the consensus networks for INBIA (a) and PERA (b) constructed by considering common predictions in all predicted cancers network (for each cancer type we take the ensemble network). Each node size in the INBIA's (a) and PERA's (b) network corresponds to collective influence and nodes are colored in red if they belong to MDS.
We further analyzed the INBIA consensus network by considering OncoPanel [12], a custom targeted next-generation sequencing assay for cancer composed by 282 genes. Only 49 genes out of 282 are present in TCPA datasets but, interestingly, the consensus network obtained in Figure S1 contains a total of 42 genes and 15 of these are also present in OncoPanel. Figure S2 represents the labeled INBIA consensus network. The complete results of this analysis are reported in Additional File 5.

3 Functional analysis

Table S7 reports gene set enrichment analysis for all genes in the TCPA dataset. Most genes related to TCPA proteins overlap with genes up-regulated by activa-

Table S4 Cancer types and best performing inference network methods with maximum F-measure value between round brackets on varying path length k.

Cancer Type	k	INBIA	PERA
BLCA	1	CLR (0.188)	ELASTICNET (0.179)
	2	MRNET (0.686)	MRNET (0.556)
	3	CLR (0.799)	CLR (0.692)
	4	CLR (0.806)	CLR (0.714
BRCA	1	GLASSO (0.186)	PLS (0.179)
	2	MRNET (0.684)	MRNET (0.550)
	3	CLR (0.805)	MRNET (0.691)
	4	CLR (0.814)	CLR (0.714)
COAD	1	CLR (0.182)	ARACNEA (0.166)
	2	MRNET (0.683)	MRNET (0.557)
	3	MRNET (0.792)	MRNET (0.690)
	4	MRNET (0.798)	MRNET (0.712)
GBM	1	PLS (0.196)	PLS (0.191)
	2	CLR (0.683)	CLR(0.546)
	3	CLR (0.811)	CLR(0.685)
	4	CLR(0.819)	CLR(0.708)
HNSC	1	PLS (0.184)	PLS (0.178)
	2	CLR (0.694)	CLR (0.563)
	3	CLR (0.808)	CLR (0.698)
	4	CLR (0.815)	CLR (0.722)
KIRC	1	PLS (0.210)	PLS (0.180)
	2	CLR (0.681)	CLR (0.564)
	3	CLR (0.796)	CLR (0.698)
	4	CLR (0.803)	CLR (0.717)
LGG	1	PLS (0.193)	PLS (0.194)
	2	MRNET (0.678)	MRNET (0.548)
	3	MRNET (0.783)	MRNET (0.673)
	4	MRNET (0.791)	MRNET (0.693)
LUAD	1	CLR (0.187)	SPEARMAN (0.188)
	2	CLR (0.690)	CLR (0.569)
	3	CLR (0.801)	CLR (0.702)
	4	CLR (0.806)	CLR (0.723)
LUSC	1	CLR (0.184)	SPEARMAN (0.184)
	2	CLR (0.691)	CLR (0.557)
	3	CLR (0.797)	CLR (0.693)
	4	CLR (0.802)	CLR (0.716)
OV	1	GLASSO (0.191)	PLS (0.174)
	2	MRNET (0.681)	MRNET (0.557)
	3	MRNET (0.785)	MRNET (0.684)
	4	MRNET (0.791)	MRNET (0.702)
PRAD	1	MRNET (0.191)	WGCNA (0.168)
	2	MRNET (0.678)	MRNET (0.556)
	3	MRNET (0.780)	MRNET (0.678)
	4	MRNET (0.788)	MRNET (0.699)
READ	1	MRNET (0.186)	CLR (0.166)
	2	MRNET (0.682)	MRNET (0.554)
	3	MRNET (0.789)	MRNET (0.687)
	4	MRNET (0.792)	MRNET (0.709)
SKCM	1	MRNET (0.188)	WGCNA (0.166)
	2	CLR (0.679)	MRNET (0.561)
	3	CLR (0.792)	MRNET (0.692)
	4	CLR (0.799)	MRNET (0.712)
STAD	1	PLS (0.179)	PLS (0.165)
	2	MRNET (0.678)	MRNET (0.552)
	3	MRNET (0.791)	MRNET (0.684)
	4	MRNET (0.797)	MRNET (0.708)
THCA	1	PLS (0.196)	PLS (0.174)
	2	MRNET (0.692)	MRNET (0.558)
	3	MRNET (0.809)	MRNET (0.687)
	4	MRNET (0.816)	MRNET (0.707)
UCEC	1	PLS (0.189)	WGCNA (0.178)
	2	MRNET (0.680)	MRNET (0.558)
	3	MRNET (0.786)	MRNET (0.689)
	4	MRNET (0.794)	MRNET (0.709)

tion of the PI3K/AKT/mTOR pathway and mediating the programmed cell death by activation of caspases [13]. Other significant enriched gene sets are hypoxia, that contain genes up-regulated in response to low oxygen levels and genes down-regulated in response to ultraviolet radiation [13]. Nodes are valuated associating their function to a measure called *node collective influence* and to the beloging of

Table S5 Cancer types contained in TCPA and the corresponding normal tissues in TissueNet v.2 and GIANT. The normal tissue counterpart was selected by checking the occurrence of cancer in that tissue in literature and the keyword was used to match the normal tissue filenames in TissueNet and GIANT.

Cancer type	Keyword	TissueNet Normal Tissues	GIANT Normal Tissues
BLCA	bladder_urothelial	urinary bladder_urothelial cells	urinary_bladder
BRCA	breast	breast	mammary_gland
COAD	colon	colon	colon
GBM	brain	brain - cerebellum<br>brain - cerebral cortex<br>brain - hippocampus<br>brain - lateral ventricle	brain
HNSC	nasopharynx	nasopharynx_respiratory epithelial cells	pharynx
KIRC	kidney	kidney	kidney
LGG	hippocampus	brain - hippocampus	hippocampus
LUAD	lung	lung	lung
LUSC	lung	lung	lung
OV	ovary	ovary	ovary
PRAD	prostate	prostate_glandular cells	prostate_gland
READ	rectum	rectum_glandular cells	rectum
SKCM	skin	skin	skin
STAD	stomach	stomach	stomach
THCA	thyroid	parathyroid gland<br>thyroid gland	thyroid_gland
UCEC	endometrium	endometrium	uterine_endometrium

Table S6 Comparison of INBIA and PERA with TissueNet. The columns report the number of predictions for a specific cancer type related to INBIA and PERA followed by the corresponding overlap with TissueNet, and the normal tissue counterpart used for the comparison. If there were multiple normal counterparts for a specific cancer, the comparison was repeated for each of them.

Cancer type	INBIA	PERA	INBIA-TissueNet	PERA-TissueNet	TissueNet Normal
BLCA	671	205	346	91	urinary bladder_urothelial cells
BRCA	405	241	193	71	breast
COAD	657	248	295	85	colon
GBM	310	275	94|113|87|79	54|74|57|43	cerebellum|cerebral cortex|hippocampus|lateral ventricle
HNSC	286	244	125	78	nasopharynx_respiratory epithelial cells
KIRC	311	234	150	94	kidney
LGG	320	294	105	75	brain - hippocampus
LUAD	703	169	326	70	lung
LUSC	670	182	311	79	lung
OV	451	283	117	63	ovary
PRAD	664	253	262	82	prostate_glandular cells
READ	658	559	288	218	rectum_glandular cells
SKCM	672	284	363	122	skin
STAD	317	259	137	91	stomach
THCA	325	262	87|131	51|69	parathyroid gland|thyroid gland
UCEC	325	205	158	76	endometrium

the node to the *minimum dominating set (MDS)*. MDS is the minimal subset of nodes such that every other node in the network that do not belong to this set is directly connected to a MDS node [14]. The proposed solution to find MDS follows a greedy approach. At each step it selects the node c with the maximum degree, sets it as 'visited' and adds it to the current MDS. Then c's neighbors, i.e. nodes directly connected to c, are set to 'visited' too and removed from the set of nodes to consider at the next step. The procedure ends when every node is visited. It has been proved that the approximation error is less than $\ln(\delta) + 2$ where δ is the maximum degree in the input graph [15]. For each tissue, the network of PPI predictions related to the best method was selected, then its MDS was computed.

The collective influence (CI) measure, first introduced in [16], finds *influencers* within a network. These nodes have a topologically key role in the spreading of information along the entire network. This can be used to stop the diffusion of an epidemic disease. The CI measure reveals the importance of 'weak' nodes that

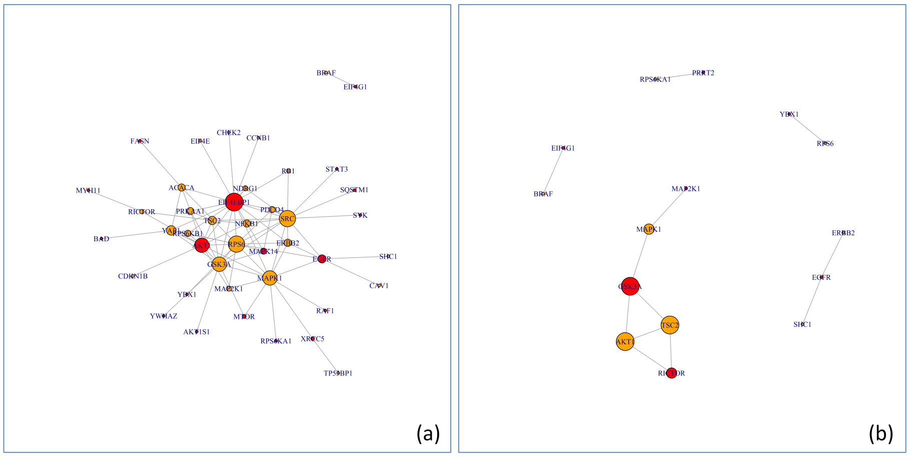

Figure S1 All cancers consensus network. These networks represent the overlap of PPIs between all best methods predictions provided by INBIA (a) and PERA(b).

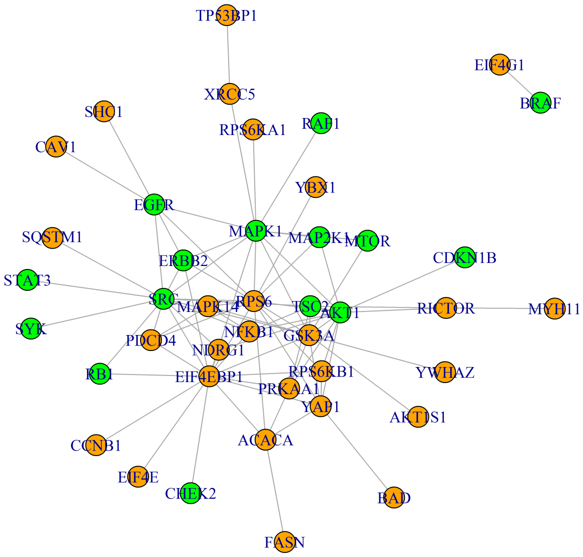

Figure S2 The INBIA consensus network obtained from the overlap of PPIs between all best methods for all cancers. The nodes in green are those present in OncoPanel.

have low degree but also takes into account the contribution of neighbors with high degree called *hubs*. In PPI networks related to a specific disease, the influencers can be proteins with a biological function related to the initiation of the signaling process for that pathology.

Table S7 Gene set enrichment analysis of all TCPA genes performed with MSigDB. The proteins are mostly involved in PI3K/AKT/mTOR pathway and apoptosis regulation. K is the number of genes in Gene Set, k the number of genes in overlap, p-value and FDR q-value are non-corrected and corrected significativity of the enrichment, respectively.

Gene Set	K	k	p-value	FDR q-value
HALLMARK_PI3K_AKT_MTOR_SIGNALING	105	20	7.25E-30	3.63E-28
HALLMARK_APOPTOSIS	161	17	6.17E-21	1.54E-19
HALLMARK_HYPOXIA	200	17	2.64E-19	4.39E-18
HALLMARK_UV_RESPONSE_DN	144	14	6.30E-17	7.88E-16
HALLMARK_APICAL_JUNCTION	200	15	2.40E-16	2.00E-15
HALLMARK_E2F_TARGETS	200	15	2.40E-16	2.00E-15
HALLMARK_MTORC1_SIGNALING	200	12	3.65E-12	2.61E-11
HALLMARK_COMPLEMENT	200	10	1.43E-09	5.96E-09
HALLMARK_ESTROGEN_RESPONSE_EARLY	200	10	1.43E-09	5.96E-09
HALLMARK_G2M_CHECKPOINT	200	10	1.43E-09	5.96E-09

Let $\delta Ball(i,r)$ for node i be the set of nodes at distance r from node i through a shortest path in graph G. Then, collective influence is:

$$CI_r(i) = (d_G(i) - 1) \sum_{j \in \delta Ball(i,r)} (d_G(j) - 1) \quad (1)$$

where $d_G(v)$ is the degree of node v in graph G. As suggested in [16], the CI for node i is more informative when $r \geq 1$ and r is lower than the diameter of the network, otherwise CI vanishes. For these reasons, we set $r = 2$.

For each tissue, the network of PPI predictions related to the best method was selected, then we computed the collective influence for each protein. The higher is the CI value the higher is the number of hubs a protein is connected to. Additional File 5 reports the lists of MDSs and collective influence of nodes, for each cancer PPI network inferred by our approach and PERA.

Author details

[1]Department of Mathematics and Computer Science, University of Catania, Viale A. Doria 6, 95125 Catania, Italy. [2]Department of Clinical and Experimental Medicine, University of Catania, c/o Dept. of Math. and Comp. Sicence, Viale A. Doria 6, 95125 Catania, Italy. [3] Department of Computer Science, University of Verona, Strada le Grazie 15, 37134 Verona, Italy.

References

1. Schäfer, J., Strimmer, K.: An empirical bayes approach to inferring large-scale gene association networks. Bioinformatics **421(6)**, 754–764 (2005)
2. Langfelder, P., Horvath, S.: Wgcna: an r package for weighted correlation network analysis. BMC Bioinformatics **9**, 559 (2008)
3. Zhang, B., Horvath, S.: A general framework for weighted gene co-expression network analysis. Statistical applications in genetics and molecular biology **4**(1), 1128 (2005)
4. Marbach, D., Costello, J., Küffner, R., Vega, N.M., Prill, R., Camacho, D., Allison, K., Kellis, M., Collins, J., Stolovitzky, G.e.a.: Wisdom of crowds for robust gene network inference. Nature methods **9**(8), 796–804 (2012)
5. Zou, H., Hastie, T.: Regularization and variable selection via the elastic net. Journal of the Royal Statistical Society: Series B (Statistical Methodology) **67**(2), 301–320 (2005)
6. Margolin, A., Nemenman, I., Basso, K.: Aracne: an algorithm for the reconstruction of gene regulatory networks in a mammalian cellular context. BMC bioinformatics **7 (Suppl 1)**, 7 (2006)
7. Faith, J., Hayete, B., Thaden, J.: Large-scale mapping and validation of escherichia coli transcriptional regulation from a compendium of expression profiles. PLoS Biology **5**(1), 8 (2007)
8. Meyer, P., Kontos, K., Lafitte, F., Bontempi, G.: Information-theoretic inference of large transcriptional regulatory networks. EURASIP journal on bioinformatics and systems biology **2007**(1), 79879 (2007)
9. Şenbabaoğlu, Y., Sümer, S., Sánchez-Vegaet, F., Bemis, D., Ciriello, G., Schultz, N., Sander, C.: A multi-method approach for proteomic network inference in 11 human cancers. PLOS Computational Biology **12**, 1–31 (2016)
10. Basha, O., Ruth, B., Sharon, M., Lerman, E., Kirson, B., Hekselman, I., Yeger-Lotem, E.: The tissuenet v.2 database: A quantitative view of protein-protein interactions across human tissues. Nucleic Acids Research **45**, 427–431 (2016)
11. Greene, C.S., Krishnan, A., Wong, A.K., Ricciotti, E., Zelaya, R.A., Himmelstein, D.S., Zhang, R., Hartmann, B.M., Zaslavsky, E., Sealfon, S.C., *et al.*: Understanding multicellular function and disease with human tissue-specific networks. Nature genetics **47**(6), 569–576 (2015)

12. Garcia, E.P., Minkovsky, A., Jia, Y., Ducar, M.D., Shivdasani, P., Gong, X., Ligon, A.H., Sholl, L.M., Kuo, F.C., MacConaill, L.E., Lindeman, N.I., Dong, F.: Validation of oncopanel a targeted next-generation sequencing assay for the detection of somatic variants in cancer. Archives of Pathology and Laboratory Medicine **141**(6), 751–758 (2017). doi:10.5858/arpa.2016-0527-OA
13. Subramanian, A., Tamayo, P., Mootha, V., Mukherjee, S., Ebert, B., Gillette, M., Paulovich, A., Pomeroy, S., Golub, T., Lander, E., Mesirov, J.: Gene set enrichment analysis: a knowledge-based approach for interpreting genome-wide expression profiles. Proceedings of the National Academy of Sciences of the United States of America **102**(43), 15545–15550 (2005)
14. Milenković, T., Memišević, V., Bonato, A., Pržulj, N.: Dominating biological networks. PloS one **6**(8), 23016 (2011)
15. Ruan, L., Du, H., Jia, X., Wu, W., Li, Y., Ko, K.: A greedy approximation for minimum connected dominating sets. Theoretical Computer Science **329**(1-3), 325–330 (2004)
16. Morone, F., Makse, H.: Influence maximization in complex networks through optimal percolation. Nature **524**, 65–68 (2015)
